# An Integrated Pest Management Intervention Improves Knowledge, Pest Control, and Practices in Family Child Care Homes

**DOI:** 10.3390/ijerph14111299

**Published:** 2017-10-26

**Authors:** Michelle Stephens, Kimberly Hazard, Debra Moser, Dana Cox, Roberta Rose, Abbey Alkon

**Affiliations:** School of Nursing, University of California, San Francisco, CA 94143, USA; kimberly.hazard@ucsf.edu (K.H.); healthy.set@hotmail.com (D.M.); danarn95060@gmail.com (D.C.); bobbie.rose@ucsf.edu (R.R.); abbey.alkon@ucsf.edu (A.A.)

**Keywords:** family child care home, integrated pest management, child care health consultant, environmental health, pesticides, pediatrics, nurse, public health, intervention

## Abstract

To reduce young children’s exposure to pesticides when attending family child care homes (FCCHs), we developed an integrated pest management (IPM) intervention for FCCH directors. First, we developed IPM educational materials and resources to provide the foundation for an IPM educational intervention for FCCHs. Next, we conducted and evaluated a six-month nurse child care health consultant (CCHC)-led education and consultation IPM intervention to increase IPM knowledge, IPM practices, IPM policies, and decrease the presence or evidence of pests. The pilot intervention study was conducted by three CCHCs in 20 FCCHs in three counties in California. Pre- and post-intervention measures were completed by the FCCH directors and observation measures were completed by the CCHCs. Results indicated significant increases in IPM knowledge, (*t*-statistic (degrees of freedom), (*t*(df) = 2.55(10), *p* < 0.05), increases in IPM practices (*t*(df) = −6.47(17), *p* < 0.05), and a 90% reduction in the prevalence of pests. There were no significant differences in changes in IPM practices based on director education, FCCH county, or IPM intervention intensity or duration. A nurse-led IPM education and consultation intervention can reduce exposures of young children attending family child care homes to harmful chemicals.

## 1. Introduction

Chronic, low-level, indoor pesticide exposures early in life are associated with adverse health outcomes, including cognitive, developmental, neurological, and respiratory problems later in life [1,2,3,4,5,6]. Two specific neurotoxic pesticides associated with children’s health problems include organophosphates (neurodevelopmental deficits and attention deficit/hyperactive disorders) [1] and pyrethroids (childhood asthma) [7]. Exposure to pests such as cockroaches, mold, mice, and dust mites have been shown to exacerbate childhood asthma symptoms, causing missed school days [5,8,9,10]. Young children are particularly vulnerable to pests and pesticides because of their rapid, organ development, including that of the brain [11,12]. It is developmentally appropriate for infants and toddlers to spend more time close to the ground where pests and pesticides accumulate [13], to engage in hand-to-mouth activity, which increases their exposure to pest and pesticide residues, and to have higher intake of air, water, and food per unit of body weight compared to adults [14]. Therefore, applications of pesticides in environments where young children spend time are potentially harmful to children’s short- and long-term health. 

Thirteen million children in the United States and one million, or approximately 63%, of children in California under six years of age attend out-of-home child care programs [15]. In the United States, licensed child care usually occurs in a child care center (CCC) [16] or a family child care home (FCCH) [17]. In 2014, there were 30,701 licensed family child care homes in California serving 312,277 children [17]. A licensed FCCH in California is run out of the director’s home, must comply with state regulations, and the licensee must have no criminal record [16]. FCCHs tend to care for mostly infants and toddlers since they have more flexible hours of care and offer a more family-friendly environment than centers [18]. Child care centers typically cost more and have higher provider to child ratios compared to FCCHs. In 2014, the average cost of full time family child care in California was $8462 per year, which was 37% less than the cost of CCC care [17]. The ages of the children in a FCCH range from young infants to school-aged children who need care after school hours [19]. FCCHs uniquely offer families a small group size and diverse schedules of care. Since FCCHs serve our youngest and most vulnerable children, FCCHs are an opportune environment to implement IPM programs. 

There are few studies of pesticides in child care centers and FCCHs in the U.S. One environmental monitoring study was conducted in 40 child care facilities, including 12 FCCHs in Northern California [20]. The study found that 90% of the facilities reported at least one pest problem and 58% had used pesticides, with 45% of those pesticides applied with broadcast application methods (e.g., sprays). This study also reported that neurotoxic pesticides, including organophosphates and pyrethroids, were routinely applied in outdoor playgrounds and classrooms in CCCs [20]. Other studies showed that pesticides applied as sprays were used in child care centers, exposing young children and staff to harmful chemicals [21,22]. These descriptive studies show that children under six years of age may be exposed to pesticides, including organophosphate and pyrethroid insecticides, while attending child care programs, in either CCCs or FCCHs. 

Integrated pest management (IPM) is an approach to managing pests that focuses on preventing infestations, monitoring pests, and limiting the use of harmful pesticides [23]. In California, the Healthy Schools Act (HSA), enacted in 2000, extended from public schools to child care centers in 2007 and amended in 2014, requires public schools and licensed child care centers to post warning signs 24 h before they use pesticides, have staff attend annual IPM trainings, provide annual reporting of pesticide use, post an IPM plan, designate an IPM coordinator, and adopt IPM practices [24]. Pesticides are used as a last resort and primarily as self-contained baits, which do not contaminate indoor or outdoor environments. The HSA AB 2865 law, designed to reduce unnecessary and potentially harmful exposures to pests and pesticides, does not extend to FCCHs [24]. 

There are a few studies of IPM interventions in child care centers. IPM interventions in child care centers have focused on teaching child care staff about IPM and providing hands-on technical assistance and follow-up consultation [25,26,27,28,29]. IPM programs in CCCs have resulted in a 50% reduction in pesticide exposure in young children [25,26,30]. Specifically, a nurse-led IPM intervention program, including educational and monthly child care health consultation, showed positive changes in staff knowledge, policies, and practices [27]. Several other CCC-based IPM intervention studies showed increases in staff’s IPM knowledge, policies, and practices [25,27,28,29,30]. An IPM train-the-trainer intervention study conducted in 892 child care programs over three years showed an increase in IPM knowledge and practices as well as a reduction in pest problems [25]. In another study of 45 child care centers in New York, Anderson et al. (2010), found that their intervention increased IPM knowledge and practices, reducing pesticide use by 44% [28]. In Indiana, schools and CCCs adopted IPM practices and reduced pesticide use after a pilot IPM program [29]. However, these studies did not include control groups. 

Child care health consultants (CCHCs) are licensed health professionals, usually nurses, trained to provide health and safety workshops, assessments, and interventions to improve the overall health and safety of the staff and children in group child care settings [31,32]. In the United States, there are national training guidelines for CCHCs, but funding for CCHCs vary state-by-state. Nurse-led child care health consultation interventions have been shown to improve health and safety policies, nutrition practices, handwashing routines, and emergency preparedness in child care centers [31,33,34,35,36]. There are no studies on IPM interventions conducted by CCHCs in FCCHs.

**Figure 1:**
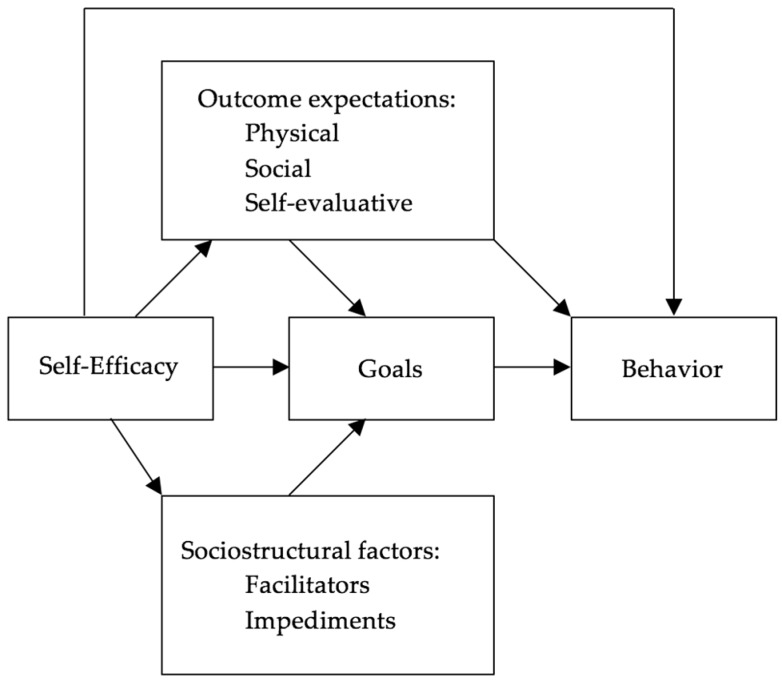
The Social Cognitive Theory Model [37].

**Figure 2:**
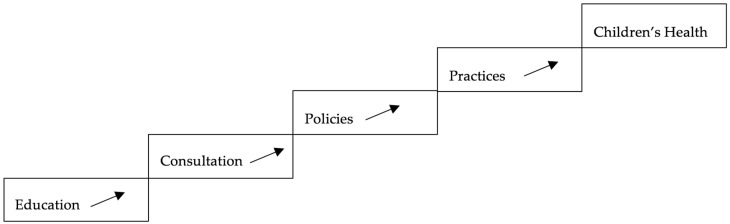
The Child Care Health Consultation Stepwise Model.

The theoretical foundations for the intervention in this study are social cognitive theory (SCT) (Figure 1), along with the Child Care Health Consultation Stepwise Model (Figure 2), which support outcomes of behavior change. The SCT framework emphasizes enhanced self-efficacy as an important driver of change. Possessing the personal belief that the child care providers or directors have the ability to solve a problem increases their motivation and perceived control to change their behavior and practices [37]. 

The SCT requires setting reasonable, achievable goals and the FCCH director and CCHC do so together by documenting the specific IPM practices they will focus on during the intervention. The Stepwise Model demonstrates a logical model for the CCHC to follow by providing educational workshops, consultation to the child care director to focus on the gaps identified during the assessments, and implementation or editing of policies that lead to changes in practices that potentially impact children’s health [38]. Interventions that have utilized nurse-led child care health consultation have used the Stepwise Model effectively [27,31,32,38]. 

Since the HSA does not apply to FCCHs in California, FCCH directors and their staff have not received the same IPM outreach as child care center providers and are more likely to be unaware of or untrained in IPM practices. California’s state regulations specify the storage of pesticides, but not the use of pesticides or pest management practices in FCCHs. Therefore, we designed an IPM project for FCCHs based on our previous work with IPM in CCCs [27]. The overall project goal was to reduce the exposures to pests and pesticides of children under six years of age attending FCCHs in Northern California in order to minimize the risk of adverse health outcomes later in life. 

This three-year IPM pilot project’s aims were: (1) to develop IPM educational materials and resources and an IPM workshop for FCCH directors; (2) to evaluate a nurse CCHC-led intervention program for FCCH directors to increase IPM knowledge, IPM practices, IPM policies, and decrease the presence or evidence of pests; and, (3) to identify if FCCH education, FCCH location (i.e., county), or CCHC intervention duration or intensity was related to improved IPM practices.

## 2. Materials and Methods 

### 2.1. Development of IPM Materials

The project’s first aim to develop IPM educational materials and resources was completed in four phases. The first phase involved conducting three focus groups, one in each county, with licensed FCCH directors to determine their awareness of IPM and their IPM needs in order to inform our staff about content to include in the IPM Toolkit for FCCHs. We recorded and transcribed the focus group discussions and then conducted a thematic analysis. 

In the second phase, our interdisciplinary team of nurses, entomologists, child care experts, and environmental health staff developed an IPM Toolkit for FCCHs. These academics, clinicians, and educators were employees of the University of California San Francisco School of Nursing, the University of California Berkeley Center for Environmental Research and Children’s Health (CERCH), the California Department of Pesticide Regulation (DPR), and the California Statewide IPM Program. The team reviewed the evidenced-based literature and best practices to develop the content of the IPM Toolkit. The Toolkit included modules describing the concept of IPM, an IPM checklist assessment, and individual pest sheet handouts that explained how to use IPM practices to address specific pests and reduce exposure to pesticides. The IPM Toolkit’s literacy level was reviewed and revised to be written at a grade five reading level. (The Integrated Pest Management Toolkit for Family Child Care Homes is available at cchp.ucsf.edu). The Toolkit was made available in English and Spanish in order to disseminate materials in the two most common languages spoken by family child care providers in California. In the third phase, we recruited two FCCHs to test the feasibility and acceptability of the IPM Toolkit and materials. The fourth phase included a meeting with an interdisciplinary IPM Alliance Team and team partners to finalize the IPM Toolkit and to develop the IPM workshop and IPM Toolbox.

### 2.2. Pilot Study Design and Sample

Next, a six-month quasi-experimental, pre/post-intervention pilot study in 20 family child care homes in three counties of Northern California was conducted from Spring 2016 to Winter 2017. A convenience sample of FCCH directors were recruited by three CCHCs. Each of the CCHCs resided in the county where they worked. The CCHCs were recruited through either the local Resource and Referral Agencies, who were funded to provide regular training and support for FCCHs, community colleges, child care planning councils, or the California Department of Social Services community care licensing public database. The FCCHs met the following inclusion criteria: small or large licensed family child care homes open to serving low-income, minority children ranging from infants to five years of age, manage their own garbage removal, speak English, and operate in one of the three participating counties. Small FCCHs are licensed to care for up to eight children, and large FCCHs are licensed to care for no more than 14 children (including children under age 10 who live in the home) [16]. The study protocol and consent form were approved by the University of California, San Francisco Institutional Review Board (IRB Study #14-14520). 

The CCHCs enrolled 23 FCCH directors and there was a 13% dropout rate resulting in 20 FCCHs who completed the study. Two FCCH directors experienced unexpected changes in their circumstances over the project period (e.g., moved, traveled away from the area). The third FCCH was located outside the study’s geographic area.

### 2.3. IPM Intervention

After the CCHCs recruited the FCCHs, they scheduled an initial visit at each FCCH to complete pre-intervention measures (i.e., demographic survey, interview, IPM checklist). The six-month intervention period started with an IPM workshop for FCCH directors and staff in English and Spanish that included a slide presentation and discussion about the health effects of harmful chemicals on children, alternatives to using pesticides, and IPM practices useful in FCCHs. A knowledge survey was completed by the attendees before and after the workshops. The IPM workshops were provided in both a group setting and individually depending on the availability of the FCCH director.

After the workshop, the CCHC met with the FCCH director to review the IPM checklist findings and to develop a six-month action plan. Then, there were monthly child care health consultations on specific issues relevant to the FCCH’s IPM needs. The CCHC documented the frequency, type (in-person, email, phone), and time required for each consultation visit. After the intervention, the CCHC conducted another interview and IPM checklist.

Each FCCH director received an IPM Toolkit in English or Spanish that included the IPM guide, IPM handouts on pests and pesticides, and the IPM checklist (cchp.ucsf.edu). The FCCH director also received an IPM Toolbox to aid in implementing IPM strategies, including ant baits, roach gel, a yellowjacket trap, a flashlight, a microfiber duster, a caulking gun with caulk, and a plastic bin for storage. Each attendee of the IPM workshop received a professional development certificate for IPM training to recognize their continuing education. 

The FCCH directors received a document compiled by the CCHCs that summarized the findings of the IPM checklist with photographs and notes to identify gaps and/or improvements in IPM practices and existing or potential pest problems specific to their FCCH. After the intervention and the post-IPM checklist was completed, the CCHCs mailed each FCCH director their post-IPM checklist summary that acknowledged the positive IPM changes they made to their FCCH as well as noting IPM practices to improve upon, and a Certification of Completion of the IPM intervention program. 

### 2.4. Measures

The IPM knowledge survey completed before and after the workshop included seven multiple choice items to assess basic knowledge of IPM, pests and pesticides, and IPM practices. Each item had one correct response and the results were summarized as the total number of correct responses. The survey was modified based on the previous IPM project in child care centers by reducing the number of questions from 10 to seven [27] and maintaining a literacy level at the fifth grade reading level. The revised knowledge survey had moderate reliability (Cronbach’s alpha, *r* = 0.74).

The demographic data of the FCCH director, program, and children were collected during the enrollment visit by the CCHC during the FCCH director’s interview. The director’s demographic characteristics included age, sex, ethnic and racial background, language spoken, level of education completed, type of child development permit held, employment status, and years of work experience. The program demographics included the size of the FCCH (small or large) and the age and number of children in attendance. The child demographics included ethnic and racial backgrounds and enrollment in government subsidy programs. Additional demographic information on the FCCH’s geographic location and community demographics was collected by the study coordinator through Google Maps and the U.S. Census Bureau data on the median income level in the FCCH’s zip code, type of geographical region of the FCCH, and type of adjacent properties to the FCCH. These are useful classes of information to determine the risk of having pest problems. 

The 30-min director interviews included open- and closed-ended questions and were conducted by the CCHC. The interview included questions on IPM knowledge, IPM policy, IPM training, use of indoor and outdoor pesticides, IPM practice, and pest problems.

The IPM checklist included 66-objective, observable items about the FCCH environment’s vulnerability to pests and the program’s IPM practices, and it was to be completed by the CCHC. Each item’s response was coded as either yes, no, or not applicable. The higher the score on the IPM checklist, the more IPM practices were met. The IPM checklist was divided into outdoor and indoor sections with six subscales. At the end of each subscale, the actual pests or evidence of pests observed were noted, prompted by a list of nine pests and a blank line to identify an “other” type of pest(s) or evidence of pests observed. A Kuder-Richardson test conducted on the pre-intervention IPM checklist had an alpha = 0.82 on 59 of the 66 items (89%) showing strong reliability. 

The CCHCs were trained on and completed the interrater reliability of the IPM checklist assessments (90%) with an experienced staff member before data collection began. The CCHCs were not blinded to the study design because they tracked the timing of assessments, workshops, and intervention activities. 

The Nurse Activity Log tracked the intensity (number of contacts), duration (number of minutes), and content of the nurse CCHC’s consultations including the date, type of consultation activity (with specific content description), recipient of activity, location of activity, travel time, preparation time, and general notes. Some of the specific consultation topics included dealing with pest infestations (mice, rats, ants, bed bugs, and spiders), hiring a pest management professional (PMP), recovering after recent flooding, implementing green cleaning, and scheduling routine house cleaning. The Nurse Activity Log was modified from a previous study of nurse CCHCs intervention in child care centers [27]. 

### 2.5. Statistical Analysis

The surveys and measures were entered into a Qualtrics^®^ database and then analyzed using IBM SPSS Statistics 24 or STATA 14.0 (StataCorp LP, College Station, TX, USA). Descriptive statistics were conducted on all measures, items, and subscales with mean ± standard deviations (SDs) for continuous variables and frequencies and percentages for categorical or nominal variables. The changes between the pre- and post-intervention measures were evaluated with paired *t*-tests for continuous data. Specific FCCH education, FCCH county, and CCHC consultation intensity and duration were analyzed in relation to IPM checklist change using chi-square analyses and *t*-tests. Statistical significance was set a priori at *p* < 0.05.

## 3. Results

The three focus groups with FCCH directors were conducted to identify what the FCCH directors knew about IPM, how they handled pest problems, and what changes were needed in our IPM Toolkit for child care centers in order to respond to their needs. The responses to the questions were analyzed thematically and there were several themes identified that informed the development of the IPM Toolkit and pest sheets (Table 1).

The demographic characteristics of the 20 FCCH directors included that all were female, all had completed some college, and they were ethnically and racially diverse (Table 2). The FCCH directors were primarily middle-aged, experienced in the child care field, and worked more than 40 h per week year-round.

The FCCHs were located in counties and regions where 9% to 35% of the children lived in poverty, with an average median household income from $34,341 to $90,364. The majority of the FCCHs were located next to private homes with only six of the 20 FCCHs (30%) having an open field on at least one side of the FCCH. 

A total of 216 children attended the 20 FCCHs. The children were from diverse racial/ethnic and socio-economic backgrounds (Table 3).

The FCCH director interviews showed that there was an increase in the number of directors who knew what IPM was from the pre-intervention (1 (5%)) to the post-intervention interview (20 (100%)). When the FCCH director was asked, “Have you ever attended a training on IPM?”, 0 (0%) answered “Yes” on the pre-interview compared to 20 (100%) on the post-interview. The FCCH directors that had a written policy for the use of pesticides went from 2 (10%) on the pre-interview to 18 (90%) on the post-interview. The FCCH directors who reported using pesticides outdoors up to six months prior to the intervention decreased from 9 (45%) to 5 (25%). 

There was an overall statistically significant improvement in director knowledge after the IPM workshop (*t*(df) = 2.55(19), *p* = 0.02) (Table 4). There was a mean ± SD increase in knowledge from 5.95 ± 1.7 to 6.9 ± 0.31 items answered correctly on the survey. 

The IPM checklist showed positive, significant changes in IPM practices from the pre- to post-intervention periods (Table 5). The IPM practices increased by 11% on the post-intervention IPM checklist compared to the pre-intervention IPM checklist. Overall, there was a statistically significant increase in the mean scores of the IPM checklist (*t*(df) = −6.47, df = 17, *p* < 0.001). The IPM checklist subscale mean scores had statistically significant increases from the pre- to post-intervention periods, except for the bathroom subscale (*p* = 0.096). The storage areas subscale showed the largest improvement with a 0.22 mean score increase from the pre- to post-intervention period. 

The FCCH directors reported 15 different types of pests as being their most common pest problem in the last six months (Table 6). The most commonly reported pest was ants (26%) followed by mice (15%), flies (10%), rats (10%), and spiders (8%). During the pre-intervention IPM checklist assessment, a total of 49 different pests were seen by the CCHCs. The post-intervention IPM checklist assessment identified five pests, which accounted for a 90% reduction in pests observed. 

The intensity of the IPM intervention for FCCH directors was a mean ± SD of 14.5 ± 3.2 contacts with the CCHC over the six-month intervention period. The majority of the CCHC contacts consisted of emails or phone calls (8.3 ± 3.0) and 4.4 ± 1.1 were in-person visits. The CCHC reviewed IPM activities 2.7 ± 0.98 times and had 5.9 ± 2.0 follow-up consultations to discuss IPM materials. The CCHC duration of consultation time with the FCCH director was a mean ± SD of 23 ± (27) min with a range of 5 to 120 min. 

There was no statistically significant relationship between the FCCH county or the FCCH director’s education and IPM checklist change scores. There was no statistically significant relationship between the CCHC consultation intensity (number of contacts) and duration (number of minutes) and IPM checklist change scores. 

## 4. Discussion

The IPM program created for FCCHs provided an IPM Toolkit with comprehensive and acceptable materials that were used in the IPM workshop and during the CCHC visits. Overall, there was a positive change in the FCCH director’s IPM awareness, knowledge, policies, practices, and pests or evidence of pests’ present after they participated in the six-month IPM intervention. The FCCH director’s education, FCCH county, CCHC consultation intensity, or CCHC consultation duration were not related to the change in IPM checklist scores. 

The FCCH directors provided feedback that the IPM Toolkit contained very useful, practical information and that the Spanish IPM Toolkit was especially unique and helpful for Spanish-speaking directors. The IPM Toolkit contained a ready-to-use sample policy that the director could immediately implement. The contents of the IPM Toolkit were also relevant and easy to share with the children’s families in order to prevent pests and the use of pesticides in the children’s homes. The IPM workshop provided in both group and one-on-one settings were effective modes of increasing IPM knowledge and practices. The IPM Toolbox indirectly acted as incentive as well as a quick and easy way to immediately implement IPM practices. One FCCH director’s husband runs a pest management business and he learned new information about IPM through this project. The directors did not realize how much more clean, healthy, and safe their home could become until after participating in the intervention. 

The pre- to post-intervention director interview showed an increase in IPM knowledge and policies and a decrease in the use of pesticides both indoors and outdoors. These results support previous IPM intervention studies that increased IPM knowledge [27,28,39], increase in IPM policies [27,28,29,39], and reduced pesticide use [25,26,28]. Pest presence decreased as reported by the FCCH directors in this project, similar to the reports of the child care directors in other IPM projects [27,28]. This IPM intervention cannot be compared to other IPM interventions in FCCHs as there are no other known IPM interventions in FCCHs. 

The IPM post-intervention checklist showed an increase in IPM practices and a decrease in the evidence and presence of pests. All of the IPM checklist subscales significantly improved post-intervention except the for bathroom subscale. The bathroom subscale had a very high initial mean score (0.95), creating a ceiling effect for improvement. The storage areas subscale showed the most improvement. Licensing requirements do not specify storage areas to be regularly cleaned and maintained; therefore, the directors may have overlooked their importance in pest management. Some items on the IPM checklist were hard to change due to the limited time of the intervention, the complexity of the maintenance problem, or the cost to repair the problem. In this study, the FCCHs had an 11% increase on the IPM checklist from pre- to post-intervention compared to an 8% increase in the IPM checklist in the study conducted in 44 CCCs in California [27]. It is possible that FCCHs are more amenable to changing IPM practices than CCCs due to their personal investment in their own home. 

The study results show that this IPM program with educational workshops combined with consultation is acceptable and feasible for FCCHs. It is interesting to note that CCHC activity (i.e., intensity and duration) was not significantly related to IPM checklist change. Yet, there was an overall, positive increase in IPM practices in this study. This finding shows the strength of the six-month IPM intervention, whose results were also not different by county or FCCH education. It is possible that fewer than six CCHC contacts could create positive change in IPM, as the CCHCs noticed most of the IPM practice changes within the first month of the intervention. This IPM program was not dependent on the individual CCHC person or county. Therefore, this IPM program may have the ability to be administered by any trained CCHC in a variety of geographic locations. This IPM program can be an effective way to decrease pests and pesticide use thereby creating a safer and healthier FCCHs. 

This project identified some differences between CCCs and FCCHs as per the previous IPM project by this team that was conducted in CCCs [27]. FCCH directors have more control over policies, facilities management, purchasing, and maintenance than CCC directors. Fewer state regulations and oversight may contribute to the increase in FCCH independence. FCCH directors are highly motivated to create change, especially with regards to issues of children’s health. They are operating out of their own home and making changes in the child care environment also means making healthy changes for their own family and home environment. FCCH directors are more difficult to recruit as their contact information is confidential, since they run their business from their own home. The attrition rate was higher than in studies in child care centers, since FCCHs are less financially stable and more dependent on one person’s ability to run their business. Scheduling in-person visits with FCCH directors is more difficult and unpredictable than with CCCs. FCCH directors are only available during small windows of time (such as naptime, evenings, or weekends). If an unexpected event happens, the FCCH director has to attend to the matter and thus cancel the CCHC visit, which increases the travel and work time for the CCHCs. Small FCCHs are particularly challenging to work with because the director is supervising children all day with no time to spend with CCHCs. Large FCCHs (>6–8 children) may have support from at least one assistant. 

### 4.1. Future Studies

When designing future intervention IPM projects in FCCHs, there are several things to consider. To facilitate recruitment and trust, include health professionals who are familiar with the family child care community. Allow the CCHC and/or intervention staff adequate time for the recruitment process to reach out to the FCCH community and over-enroll 10% or 20% FCCHs to allow for the expected dropout. Be flexible and creative when communicating with FCCH directors; identify the directors preferred means of communication—email, phone, postal service, or text message. The intervention period could be less than six months but our findings can only be generalized for six-month interventions conducted by trained nurse-CCHCs. Creating the IPM Workshop as a free, online workshop in English and Spanish will aid in widely disseminating IPM information. Collect specific information on types and frequency of pesticide exposure by including objective measures of pesticides and identifying pesticides used on-site. For example, dust samples could identify the specific pesticides present in a child care center. The IPM checklist could include a review of the pesticide products observed on-site by including a list of the product names, label information, and storage location. It would be interesting to further explore how the FCCH’s geographic location affects pest presence and pesticide use and exposure. There may be an increase in pest and pesticide exposure if a home is surrounded by open fields, agriculture, or restaurants compared to other residential homes or buildings. 

### 4.2. Limitations

Although this study showed some positive changes in IPM after the intervention, there are several limitations. This was a small, quasi-experimental pilot study that did not include a control or comparison group of FCCHs. There was no objective research assistant to conduct the IPM checklist assessments; rather, the CCHC completed both the assessments and intervention which may have introduced bias in the results. Since there are no known IPM intervention studies in FCCHs, we can only compare our findings with IPM interventions in CCCs. 

## 5. Conclusions

Family child care is a unique, intimate care setting where there is a close relationship with the families and communities they serve. FCCH directors invest an incredible amount of their lives and livelihood in the care and development of young children. FCCHs can be one of the best ways to access families with young children. This six-month education and consultation intervention, where FCCH directors received a one-hour IPM Workshop, an IPM Toolbox, an IPM Toolkit, and monthly consultations specific to each FCCH creates positive, significant change in IPM awareness, knowledge, practices, and policies. 

Interventions to increase the use of IPM in FCCHs require interdisciplinary teams, including nurses, public health professionals, entomologists, housing agencies, environmental experts, and child care experts. The FCCH IPM Toolkit is available online in English and Spanish (cchp.ucsf.edu) to assist providers and health professionals develop IPM interventions. IPM interventions in FCCHs can reduce the exposure of young children to harmful chemicals and pesticides and prevent health problems in the future. Family child care home providers are an important target audience for IPM interventions because they serve the largest number of our youngest and most vulnerable ethnically-diverse, low-income families in primarily under-resourced communities.

## Figures and Tables

**Table 1:** Focus Group Themes on IPM Needs and Practices.

Question	Summary
What are the most frequent pests you’ve seen inside and outside your FCCH in the past 6 months? 12 months? Ever?	Flies, fruit flies, and gnats Rodents such as mice, rats, and gophers Ants and spiders (fear of spiders was more common than actual encounters or bites)
What do you do to get rid of pests in your FCCH?	FCCHs in each county in our sample had different ways to deal with pests: search for web-based home remedies, hire pest control companies, and shop at stores for pesticide sprays General cleaners and disinfectants were used to eliminate pests Varying levels of understanding of what a pesticide is (e.g., pesticides were associated with farm fields, not indoor pesticide products), and of toxicity (e.g., pesticide sprays were considered safe once dried)
Where do you find information to how to handle pest problems?	FCCHs in two out of three counties relied on online searches Across all counties, FCCH directors put a lot of trust into “perceived” pest experts or professionals (PMPs, home remedy generators, commercial store employees)
What’s the best way to share new information with other FCCH directors?	A variety of family child care intermediaries (associations, resource and referral agencies, Head Start agencies, school districts) FCCHs in two out of three counties used electronic information, but those in another county overwhelmingly did not use computers and did not have email addresses
IPM Toolkit Feedback	Positive feedback on the toolkit; overall it was well-liked Durability was a benefit Wall space in FCCHs is limited, so informational posters wouldn’t be ideal We received ideas for new pest and fact sheets Ability to make photocopies for parents was important
Other	All were excited to share information with families

**Table 2:** FCCH Director Demographics, *N* = 20.

**Demographic Characteristic**	***N*** **(%)**
Gender	
Female	20 (100%)
Race/Ethnicity	
White	9 (45%)
Latino	5 (25%)
Black, Asian, or Multi-racial	6 (30%)
Education	
Some college/Associate’s Degree	13 (65%)
Bachelor’s Degree	4 (20%)
Master’s Degree or Higher	3 (15%)
**Demographic Characteristic**	**Mean (SD)**
Age	47.4 (14.6)
Years worked in child care	15.65 (11.2)
Hours worked per week	53.9 (8.2)
Months worked per year	11.6 (1.3)

**Table 3:** FCCH Child Demographics, *N* = 20.

**Demographic Characteristic**	***N*** **(%)**
Race/Ethnicity	
White	78 (36%)
Latino	62 (29%)
Black	29 (13%)
Asian	13 (6%)
Mixed/Other	34 (16%)
Total Children in FCCHs	216 (100%)
**Demographic Characteristic**	**Mean (SD)**
Number of children	10.8 (4.3)
Children receiving gov’t or another subsidy	8.2 (6.0)
Full-Time children	6.7 (3.7)
Part-Time children	4.8 (4.4)
Children living in home	1.7 (0.6)
Children < 1 year old	1.0 (0.6)
1-year-old	1.9 (1.0)
2-year-old	2.9 (2.4)
3-year-old	2.6 (1.9)
4-years-old through Pre-K	2.6 (1.2)
School-age	3 (2.7)

**Table 4:** IPM Workshop Knowledge Survey Results, *N* = 20.

Pre- and Post-Workshop Knowledge Survey Questions	Pre-Workshop		Post-Workshop	
	Correct *N* (%)		Correct *N* (%)	
1. IPM keeps pests out while reducing the use of pesticides	18	90%	19	95%
2. Pests need food, water, and shelter to survive	17	85%	20	100%
3. Mold can trigger asthma	15	75%	19	95%
4. Bait stations are the pesticide with least health risk	15	75%	20	100%
5. Cockroaches can live in cardboard boxes	19	95%	20	100%
6. Keep food in containers with tight-fitting lid—IPM indoor practice	18	90%	20	100%
7. Prevent pests from entering FCCH by sealing cracks and crevices	17	85%	20	100%
	Mean (SD)		Mean (SD)	
	5.95 (1.70)		6.9 (0.31)	

**Table 5:** IPM Checklist: Subscale and Total Mean by Pre- and Post-Intervention.

Subscales	PRE-Intervention	POST-Intervention	Paired *t*-Test (df), *p*
	Mean (SD)	Mean (SD)	
Outdoor			
Garbage, Recycling, and Compost	0.86 (0.21)	0.98 (0.06)	−2.63 (19), 0.016 *
Buildings: Structure, Landscaping, and Play Area	0.72 (0.19)	0.86 (0.13)	−3.14 (12), 0.008 *
Indoor			
Kitchen and Eating Area	0.83 (0.17)	0.95 (0.12)	−3.3 (19), 0.004 *
Bathroom	0.95 (0.09)	0.98 (0.08)	−1.75 (19), 0.096
Living and Play Areas	0.91 (0.10)	0.97 (0.07)	−2.84 (17), 0.011 *
Storage Areas: Attic, Basement, Garage, or Shed	0.69 (0.29)	0.91 (0.14)	−3.85 (17), 0.001 *
Total Checklist Score	0.82 (0.12)	0.93 (0.09)	−6.47 (17), 0.000 *

* Statistically significant at *p* < 0.05.

**Table 6:** Type and Number of Pests Observed During IPM Checklist Assessment.

Pest	Total # PRE	Total # POST	% ∆
Ants	7	2	71%
Cockroaches	0	0	-
Fleas	0	0	-
Flies	5	0	100%
Spiders	21	3	86%
Mosquitoes	0	0	-
Yellowjackets	3	0	100%
Rats/Mice	5	0	100%
Snails/Slugs	1	0	100%
Other	2	0	100%
Total	49	5	90%
